# Biological Activities, Health Benefits, and Therapeutic Properties of Avenanthramides: From Skin Protection to Prevention and Treatment of Cerebrovascular Diseases

**DOI:** 10.1155/2018/6015351

**Published:** 2018-08-23

**Authors:** Andrea Perrelli, Luca Goitre, Anna Maria Salzano, Andrea Moglia, Andrea Scaloni, Saverio Francesco Retta

**Affiliations:** ^1^Department of Clinical and Biological Sciences, University of Torino, Orbassano, Torino, Italy; ^2^CCM Italia, Torino, Italy; ^3^Proteomics & Mass Spectrometry Laboratory, ISPAAM, National Research Council, Napoli, Italy; ^4^Plant Genetics and Breeding, Department of Agriculture, Forest and Food Sciences, University of Torino, Grugliasco, Torino, Italy

## Abstract

Oat (*Avena sativa*) is a cereal known since antiquity as a useful grain with abundant nutritional and health benefits. It contains distinct molecular components with high antioxidant activity, such as tocopherols, tocotrienols, and flavanoids. In addition, it is a unique source of avenanthramides, phenolic amides containing anthranilic acid and hydroxycinnamic acid moieties, and endowed with major beneficial health properties because of their antioxidant, anti-inflammatory, and antiproliferative effects. In this review, we report on the biological activities of avenanthramides and their derivatives, including analogs produced in recombinant yeast, with a major focus on the therapeutic potential of these secondary metabolites in the treatment of aging-related human diseases. Moreover, we also present recent advances pointing to avenanthramides as interesting therapeutic candidates for the treatment of cerebral cavernous malformation (CCM) disease, a major cerebrovascular disorder affecting up to 0.5% of the human population. Finally, we highlight the potential of foodomics and redox proteomics approaches in outlining distinctive molecular pathways and redox protein modifications associated with avenanthramide bioactivities in promoting human health and contrasting the onset and progression of various pathologies.

The paper is dedicated to the memory of Adelia Frison

## 1. Introduction

Oats are cereal grain crops belonging to the family of Poaceae (or Gramineae) [1]. Two main species of oat grow naturally, namely, *Avena sativa* and *Avena nuda*. The former, known as common oat, is the most widely cultivated, especially in the cool and moist regions of Northern Europe and North America [2]. Among the common cereal grains, oats are consumed at lower rates than wheat and rice all over the world. However, dietary fiber content, nutritional value, and health benefits of oats are high. Indeed, the increasing interest of consumers towards whole grain oats is mainly driven by its advantageous composition in macronutrients: (i) lipids with a high degree of unsaturation, including oleic and linoleic acids (about 40% and 36% of total fatty acids, resp.), (ii) proteins with a favorable composition of essential amino acids, and (iii) dietary fibers with a high content of *β*-glucan (2–8.5% w/w of oat seed). In particular, the high levels of *β*-glucan present in oats have been shown to contribute in reducing total plasma concentration of cholesterol and low-density lipoprotein (LDL) cholesterol, the main risk factors for coronary heart disease (CHD).

A growing body of evidence suggests that oats contain other important bioactive compounds, such as phenolic compounds, which exert protective effects against the development of various pathologies, including cardiovascular diseases (CVDs), diabetes, inflammatory bowel disease (IBD), cancer, obesity, and celiac disease, acting synergistically with dietary fibers [3]. Phenolic compounds are major secondary products of plant metabolism, consisting of at least one aromatic ring bearing one or more hydroxyl groups (phenolic unit). Their chemical structure may range from that of a simple phenolic molecule (phenolic acids) to that of a complex high molecular weight polymer (polyphenols). Depending on the number and type of phenolic units, they can be divided into at least 10 different molecular classes, including simple phenols (phenolic acids), and intermediate (e.g., flavonoids and anthocyanins) and high (e.g., stilbenes, coumarins, and tannins) molecular weight polyphenols. From the biological point of view, phenolic compounds have been show to possess numerous activities, the most important being the antioxidant activity, which prevents lipid peroxidation and cellular oxidative damage mediated by harmful free radicals [4, 5]. This property is related to the ability of phenolic compounds to scavenge free radicals, donate hydrogen atoms or electron, or chelate metal cations, which is dictated mainly by the number and position of the hydroxyl groups and the nature of substitutions on the aromatic rings, as demonstrated by structure-activity relationship analyses. More in general, phenolic compounds have been involved in cellular defense against various stressful events [2, 6, 7] and shown to possess several health-promoting properties [8], including prophylactic activity against arteriosclerosis, CVDs, inflammatory processes, and certain forms of cancer [9, 10].

**Figure 1:**
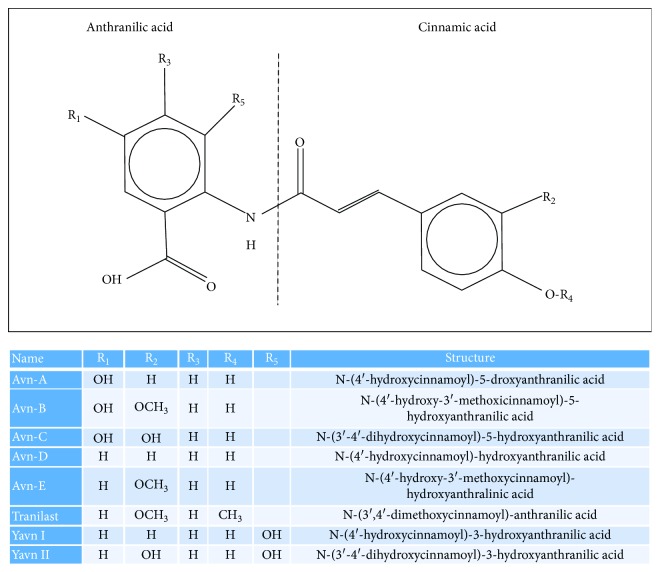
Chemical structure and names of some natural (Avn), synthetic (Tranilast), and recombinant (YAvn) avenanthramides. Avns are low molecular weight phenolic compounds consisting of an anthranilic acid linked to a hydroxycinnamic acid with an amide bond. Different forms of Avns have been either extracted from oats, produced by chemical synthesis, or generated by recombinant DNA techniques in yeast cells.

The type and concentration of phenolic compounds in whole-grain cereals are influenced by the plant variety and grain nature. In particular, besides containing high levels of phenolic acids, tocopherols, and alk(en)ylresorcinol derivatives, oats are a unique source of avenanthramides (Avns; also known as N-cinnamoylanthranilate alkaloids or anthranilic acid amides), which are not present in other cereals [11]. Avns are low molecular weight phenolic amides consisting of an anthranilic acid linked to a hydroxycinnamic acid with an amide bond (Figure 1). They were originally identified as phytoalexins produced by the plant in response to exposure to pathogens, such as fungi [12, 13]. Oats contain a unique group of approximately 40 different types of Avns, which are present in both oat grains and leaves [14–16]. The most abundant are Avn-A (N-(4′-hydroxycinnamoyl)-5-hydroxyanthranilic acid), Avn-B (*N*-(4′-hydroxy-3′-methoxycinnamoyl)-5-hydroxyanthranilic acid), and Avn-C (*N*-(3′-4′-dihydroxycinnamoyl)-5-hydroxyanthranilic acid) (Figure 1), which are amides of 5-hydroxyanthranilic acid with *p*-coumaric, ferulic, and caffeic hydroxycinnamic acids, respectively [13, 17, 18]. These Avns are constitutively expressed in the kernel, reaching the highest concentration in the bran, and appear in almost all milling fractions [17]. A number of studies demonstrate that these natural products have strong antioxidant activity both *in vitro* and *in vivo*, as well as anti-inflammatory, anti-itching, anti-irritant, antiatherogenic, and antiproliferative activities, which may prevent or limit cellular oxidative dysfunctions and the development of oxidative stress-related diseases, such as neurodegenerative and cardiovascular diseases, and provide additional protection against skin irritation, aging, CHD, and cancer [16, 17, 19–32].

Apart from natural compounds isolated from oats, avenanthramide analogs endowed with important biological properties have been artificially produced by organic synthesis methodologies, including the pharmaceutical drug Tranilast™ (N-[3′,4′-dimethoxycinnamoyl]-anthranilic acid; Rizaban, Kissei Pharmaceutical Co., Japan), which is currently used in Japan and South Korea as an antihistamine to treat bronchial asthma, atopic dermatitis, keloids and hypertrophic scars, allergic conjunctivitis, allergic rhinitis, and other allergic disorders [33–35]. Notably, whereas several years of clinical use have established that Tranilast has very low adverse effects and good toleration by patients, the beneficial effects of this drug have also been seen in a variety of other pathologies, such as scleroderma and other skin diseases related to excessive fibrosis, cancer, diabetes, and autoimmune, cardiovascular, and renal diseases [36]. Tranilast efficacy has been mainly attributed to its capacity to inhibit the release of proinflammatory factors from leukocytes, including mast cells and macrophages, and suppress collagen deposition, and has been associated mainly with the inhibition of the TGF-*β* pathway, although this drug affects other pathways as well [37, 38].

Besides natural and synthetic Avns, novel Avn analogs have been produced in recombinant yeast, including N-(4′-hydroxycinnamoyl)-3-hydroxyanthranilic acid (YAvn I) and N-(3′-4′-dihydroxycinnamoyl)-3-hydroxyanthranilic acid (YAvn II), which were generated by engineering a *Saccharomyces cerevisiae* strain with two plant genes (*4*cl*-2* from tobacco and *hct* from globe artichoke) encoding key proteins involved in the biosynthesis of phenolic esters [39]. Remarkably, YAvn I and YAvn II share structural similarity with Avn-A and Avn-C, respectively (Figure 1), and were shown to possess bioactive properties relevant to biomedical applications, including potent antioxidant, anti-inflammatory, and antiproliferative properties. Indeed, they were effective in rescuing major pathological phenotypes in both cellular and animal models of Cerebral Cavernous Malformation (CCM) disease, a human cerebrovascular disorder of genetic origin implicating oxidative stress and inflammation as main pathogenetic events [40, 41].

## 2. Radical-Scavenging and Antioxidant Activity of Avenanthramides

The antioxidant activity of oat components was initially suggested by the evidence that oat flour could be used as a food preservative from oxidative deterioration due to its ability in retarding the initial peroxide formation and rancidity [42, 43]. Subsequently, Lingnert and coworkers originally determined the antioxidative capacity of *N*-(4′-hydroxy-3′-methoxy-(*E*)-cynnamoil)-5-hydroxyanthranilic acid and *N*-(4′-hydroxy-3′-methoxy-(*E*)-cynnamoil)-5-hydroxy-4-methoxyanthranilic acid in oxygen consumption experiments with a linoleic acid-based system [44]. The antioxidant properties of oat extracts and their components, including avenanthramides, were eventually demonstrated directly by assaying purified compounds from different oat cultivars [45, 46]. In particular, Emmons and colleagues examined oat milling fractions to determine their potential as dietary antioxidants, showing that three avenanthramide isoforms (Avn-A, Avn-C, and Avn-K) were among the most important oat metabolites endowed with antioxidant activity [46]. Then, Peterson and coworkers synthesized the three major oat avenanthramides (Avn-A, Avn-B, and Avn-C) and tested their antioxidant activities using two *in vitro* assays, such as the inhibition of beta-carotene bleaching and the reaction with the free radical 2,2-diphenyl-1-picrylhy-drazyl (DPPH), demonstrating that Avn-C has greater antioxidant activity than Avn-B and Avn-A [47].

The antioxidant properties of Avns were also investigated in *in vivo* models. In particular, Avn-C supplementation in the diet of rats at a concentration of 0.1 g/kg was effective in reducing reactive oxygen species (ROS) levels in the soleus muscle. Moreover, Avn-C-fed rats had higher superoxide dismutase activity in the vastus lateralis muscle (DVL), liver, and kidney, and higher glutathione peroxidase activity in the heart and DVL, compared to control rats. In addition, Avn-C supplementation attenuated the increased ROS production in the soleus and lipid peroxidation in the heart induced by exercise [19].

The bioavailability of Avns was examined by Chen and coworkers in hamsters [22], where it was observed that plasma concentration of Avns and phenolic acids peak at 40 min after the animals were gavage with saline containing 0.25 g oat bran phenol-rich powder. While *p*-coumaric acid was the most bioavailable among oat phenolics, Avn bioavailability appeared very low, probably due to metabolite distribution in other tissues and the corresponding biotransformation rate. The same authors also investigated the bioavailability and antioxidant action of major Avns, including Avn-A, Avn-B, and Avn-C, in humans [21]. At doses of 0.5 and 1.0 g of an Avn-enriched mixture (AEM), Avns reached the maximum peak in plasma at 1.5 and 2.3 h, respectively. Avn-A and Avn-B bioavailability was 18- and 5-fold higher in humans than in hamsters, respectively. Interestingly, consumption of Avn-enriched oat extracts significantly increased the plasma concentration of reduced glutathione (GSH), the body's master antioxidant. Specifically, after consumption of 0.1 g of AEM, plasma GSH levels increased 21% from baseline at 15 min, without apparent adverse side effects [21]. Moreover, Avn-rich extract from oat was also reported to possess an effective antioxidant activity against D-galactose-induced oxidative stress [48]. Furthermore, it was demonstrated that Avns, including Avn-A, significantly increased heme oxygenase-1 (HO-1) expression in HK-2 cells in both dose- and time-dependent manners, showing that this effect involved ROS production and Nrf2 nuclear translocation [49, 50].

The Avn analog Tranilast was also reported to be effective in reducing the generation of ROS, including hydrogen peroxide (H_2_O_2_) and hydroxyl radical (OH^•^), suggesting potential clinical applications [51]. However, the mechanisms of its antioxidant activity are yet to be clarified.

On the other hand, Avn analogs produced in recombinant yeast, including YAvn I and YAvn II, were originally shown to have strong antioxidant activity when tested in an ABTS^•+^ radical quenching assay, as well as the capacity to reduce intracellular ROS levels in a cellular model of CCM disease, as evaluated with a cellular antioxidant assay [39]. Subsequent *in vitro* studies demonstrated that YAvn I and YAvn II positively regulate cell antioxidant defense mechanisms through the upregulation of forkhead box protein O1 (FOXO1) and superoxide dismutase 2 (SOD2) expression levels [40]. In addition, recent studies in an animal model of CCM disease have extended these findings, demonstrating the effectiveness of YAvns in major oxidative stress-related disease phenotypes [41] (Table 1).

## 3. Anti-Inflammatory Activity of Avenanthramides

The ancient literature already described the anti-inflammatory and anti-itching properties of oat extracts. In fact, Greek and Latin literatures report the use of oatmeal as topical therapy for a variety of dermatological conditions [29]. Since 1945, several studies showed the benefits of colloidal oatmeal bath as soothing treatment as well as nonirritating, cleansing formulation for inflamed, itchy skin associated with various xerotic dermatitis [16, 29]. Despite widespread use for skin irritation, the phytochemicals present in oat and responsible for the anti-inflammatory activity were not defined until 2004. Liu and colleagues first reported the potential anti-inflammatory and antiatherogenic properties of Avn-enriched extracts of oats, which inhibited the IL-1*β*-stimulated endothelial cell secretion of proinflammatory cytokines (IL-6) and chemokines (IL-8 and MCP-1), as well as expression of adhesion molecules (ICAM-1, VCAM-1, and E-selectin) and adhesion of monocytes to endothelial cell monolayer [29]. Similarly, CH_3_-Avn-C, a synthetically prepared methyl ester derivative of Avn-C, significantly and dose-dependently decreased mRNA expression and secretion of IL-6, IL-8, and MCP-1 in endothelial cells and inhibited IL-1*β*- and TNF*α*-stimulated NF-*κ*B activation by preventing the phosphorylation of I*κ*B kinase and I*κ*B [26]. Moreover, Sur and colleagues found that keratinocites treated with Avns displayed a significant inhibition of TNF-induced NF-*κ*B activity and subsequent reduction of IL-8 release, suggesting that oat Avns may have putative anti-itching activity [16]. Furthermore, dihydro-avenanthramide D (DH Avn-D), a synthetic analog of avenanthramide, was shown to inhibit mast cell degranulation and exhibit anti-inflammatory effects through the activation of the neurokinin-1 receptor [52]. In addition, avenanthramide supplementation was able to attenuate exercise-induced inflammation in postmenopausal women by reducing neutrophil respiratory burst activity, plasma C-reactive protein and IL-1*β* levels, and NF-*κ*B activation in peripheral blood mononuclear cells [53]. Notably, differences in the ability to inhibit NF-*κ*B among Avns have been ascribed to molecular structural variations [54].

On the other hand, the anti-inflammatory properties of the Avn analog Tranilast have been attributed mainly to its capacity to inhibit the release of chemical mediators from mast cells and basophils [34, 55], suppress COX-2 and iNOS expression [56], and limit TNF*α*-induced secretion of IL-6 and surface expression of vascular adhesion molecules, including VCAM-1, ICAM-1 and E-selectin, in endothelial cells by inhibiting NF-*κ*B-dependent gene transcription [38]. Furthermore, Tranilast anti-inflammatory properties were shown to be triggered by the induction of HO-1 expression via ERK1/2 activation [55]. Finally, recombinant YAvns were demonstrated to downregulate NF-*κ*B and rescue inflammatory phenotypes in cellular and animal models of CCM disease [41] (Table 2).

## 4. Antiproliferative Activity of Avenanthramides

Clear evidence demonstrates that Avns, including Avn-C and its methylated derivative (CH_3_-Avn-C), can significantly inhibit proliferation of distinct cell lines, such as human colon and breast cancer cells, and vascular smooth muscle cells (VSMC) [25, 31, 57]. Indeed, it has been reported that Avns induce cell cycle arrest at the G1 phase by upregulating the p53-p21cip1 pathway and inhibiting phosphorylation of the retinoblastoma protein (pRB) [25], and may activate apoptosis [57]. Moreover, methylated Avn-C was shown to inhibit proteasome activity and increase the levels of ubiquitin-conjugated proteins in endothelial cells, suggesting that inhibition of proteasome activity and consequent stabilization of the p53 protein are a plausible mechanism underlying the inhibitory effect of Avns on the cell cycle [25]. In particular, by testing the antiproliferative effect of Avns on distinct cancer cell lines, it was found that Avn-enriched oat extracts, Avn-C, and the methyl-ester derivative of Avn-C were more effective on colon cancer cell lines, including CaCo-2, HT29, LS174T, and HCT116 cells, than on prostate or breast cancer cell lines [25]. Furthermore, the synthetic DH Avn-D was shown to inhibit human breast cancer cell invasion through inhibition of MAPK/NF-*κ*B and MAPK/AP-1 pathways and suppression of MMP-9 expression [32]. In addition, the Avn analog Tranilast was shown to exert inhibitory effects on proliferation, epithelial-mesenchymal transition (EMT), and invasion of cancer cells [58]. Moreover, it was reported to inhibit proliferation, chemotaxis, and tube formation of human microvascular endothelial cells *in vitro* and angiogenesis *in vivo* [59], as well as vascular endothelial growth factor (VEGF)-induced vascular permeability [60], suggesting that it might ameliorate angiogenesis-related diseases, such as tumor metaplasia, rheumatoid arthritis, diabetic retinopathy, and age-related macular degeneration, acting as a novel angiogenesis inhibitor [59, 61, 62]. Finally, recent evidence demonstrates that recombinant YAvn I and YAvn II are endowed with stronger antiproliferative properties than natural Avns, including Avn-B, due to their enhanced capacity of reducing intracellular ROS levels and cyclin D1 expression [40] (Table 3).

## 5. Therapeutic Benefits of Avenanthramides

There is compelling evidence that oxidative stress plays a major role in the pathogenesis and progression of major human diseases, including atherosclerosis, diabetes, inflammatory diseases, cardiovascular diseases, cancer, and neurological disorders, such as amyotrophic lateral sclerosis, Alzheimer's (AD) and Parkinson's (PD) diseases [63], and is also implicated in aging [64].

Oxidative stress occurs either when excess ROS are produced in cells, which could overwhelm the normal antioxidant capacity, or upon impairment of antioxidant defense mechanisms. ROS toxicity contributes to protein, lipid and DNA damage, inflammation, cell and tissue injury, and apoptosis. Nevertheless, ROS also play important physiological functions, whereas emerging evidence demonstrates that the biological impact of ROS depends not only on their intracellular levels and rate of formation and decay but also on their chemical nature and subcellular localization [65, 66]. Thus, inappropriate removal of ROS by antioxidants may cause paradoxical reductive stress and thereby induce or promote disease [63, 67, 68].

Due to their capacity to scavenge ROS and prevent oxidative stress, antioxidants (including natural and synthetic phenolic compounds) have long been credited with helping to live longer and stay healthier, and looked upon as effective therapeutic options for prevention and treatment of various oxidative stress-related diseases. Natural antioxidants are primarily phenolics that may occur in all parts of plants [69]. Specifically, beneficial effects on human health of phenolic compounds with high antioxidant properties obtained from oats have been reported in many studies and shown to protect cells against oxidative damage [23, 70]. Furthermore, several compositions containing oat Avns or derivatives have been described in pharmaceutical patents for use in cosmetic, nutraceutical, and therapeutic preparations due to their antioxidant, anti-inflammatory, anti-itching, antiallergic, antihistaminic, antiasthmatic, and antiaging activities. In particular, the synthetic drug Tranilast has been approved since 1982 in Japan and South Korea and, as mentioned above, is currently used as an antihistamine to treat bronchial asthma, atopic dermatitis, allergic conjunctivitis, allergic rhinitis, and other allergic disorders, with indications for keloids and hypertrophic scars, scleroderma, and other skin disease related to excessive fibrosis [36]. In addition, it was proposed for treatment of autoimmune diseases, such as arthritis and multiple sclerosis, and as an inhibitor of angiogenesis [37, 71]. Moreover, the high potential of Tranilast in inhibiting pathological cellular growth processes, such as tumor-related ones, was investigated with promising results [37, 58, 72–81].

On the other hand, a randomized, placebo-controlled, double-blind pilot study, led to determine whether the Avn-enriched bran reduces biomarkers of inflammation, demonstrated that consuming Avns in a whole food form, that is, Avn-enriched oat bran, may affect specific biomarkers of inflammation in older, overweight, or obese adults [82]. Considering the anti-inflammatory properties of Avns and their capacity to inhibit smooth muscle cell proliferation and increase NO production, these compounds were proposed for prevention or therapy of atherosclerosis and associated cardiovascular diseases. Data also pointed to the potential benefit of including oats and oat bran in daily meals over the long term [26]. Interestingly, recent evidence highlighted the combined antioxidant, anti-inflammatory, and anticancer effects of individual synthetized Avns and a mixture of natural Avns on CaCo-2 and Hep3B cancer cells, showing that both natural and synthetic Avns activate caspases 2, 8, and 3 and downregulate hTERT, MDR1, and COX-2 genes, and suggesting that oat-based foods fortified with Avns could be an alternative to produce functional foods with major health benefits [83]. Furthermore and importantly, recent findings demonstrated that both Tranilast and YAvns were effective in rescuing prooxidant and proinflammatory phenotypes associated with CCM disease, a cerebrovascular disorder associated with altered redox homeostasis and signaling and enhanced susceptibility to oxidative stress and inflammatory insults, thus widening the therapeutic potential of these compounds [41].

## 6. Avenanthramides as Potential Therapeutics for Cerebral Cavernous Malformation Disease

CCM, also known as cavernous angioma or cavernoma, is a major cerebrovascular disease characterized by clusters of abnormally dilated and leaky capillaries occurring in brain, spinal cord, and retina, with a prevalence of 0.3–0.5% in the general population. These vascular anomalies, referred to as CCM lesions, can be single or multiple (up to hundreds), as detected by magnetic resonance imaging, and may result in severe clinical symptoms at any age, including recurrent headaches, focal neurological deficits, seizures, stroke, and intracerebral hemorrhage (ICH) [84]. CCM disease has proven genetic origin (OMIM 116860), being caused by loss-of-function mutations in three genes, *KRIT1* (*CCM1*), *CCM2*, and *PDCD10* (*CCM3*). It may arise sporadically or is inherited as autosomal dominant condition with incomplete penetrance and highly variable expressivity even among members of the same family, including wide differences in lesion number, size, and susceptibility to ICH [84–86]. Despite significant recent advances in our understanding of the pathophysiology of CCM disease, no direct therapeutic approaches are available so far, besides the surgical removal of accessible lesions [84, 87].

Accumulated evidence demonstrates that loss-of-function mutations of CCM genes have pleiotropic effects on several redox-sensitive molecules and mechanisms that control cellular homeostasis and defenses against oxidative stress and inflammation, thereby sensitizing cells to local oxidative stress and inflammatory events [84, 86, 88–95]. In particular, KRIT1 loss-of-function has been shown to affect major antioxidant pathways and mechanisms, including the FOXO1-SOD2 axis and the Nrf2 antioxidant pathway [89, 94], and the autophagic degradation of dysfunctional, ROS-generating mitochondria [89, 91]. On the other hand, there is emerging evidence that Avns, including YAvns, can enhance cellular defenses against oxidative stress by inhibiting the activity of prooxidant and proinflammatory proteins, such as NADPH oxidase and NF-*κ*B [41], and stimulating the upregulation of antioxidant molecules, such as GSH and SOD2 [21, 40]. Indeed, treatment of KRIT1-knockout and KRIT1-silenced cellular models with YAvns was effective in reverting molecular phenotypes caused by KRIT1 loss-of-function, including the downregulation of FOXO1 and SOD2 and the upregulation of cyclin D1 [40]. Furthermore, both YAvns and Tranilast were able to induce a rescue of major phenotypic signatures in a mouse model of CCM disease, including altered redox homeostasis and signaling, destabilized endothelial cell-cell junctions and blood-brain barrier, enhanced vascular permeability, and reduced susceptibility to oxidative stress and inflammatory insults, suggesting potential therapeutic benefits for CCM disease [41]. Further studies aimed at a comprehensive characterization of the pleiotropic effects and mechanisms of action of natural and recombinant Avns will provide useful insights into these and other promising therapeutic benefits.

## 7. Avenanthramide and Aging Processes: A New Elixir of Youth?

Oatmeal has been used for centuries as a soothing agent to relieve itch and irritation associated with various xerotic dermatoses. Today, it is available in various dosage forms from powders for the bath to shampoos, shaving gels, and moisturizing creams, and has been approved as a skin protectant by the US Food and Drug Administration (FDA) [27].

Among oat constituents, Avns are known to suppress histamine release at very low doses, helping to plump up the skin, reduce wrinkles, and restore the skin natural barrier. Indeed, oat Avns have been shown to represent the main group of active polyphenolic antioxidants responsible for oatmeal anti-inflammatory, antierythema (antiredness), antipruritic (anti-itching), and antihistaminic properties. Consistently, several studies have demonstrated their benefits in reducing eczema and other inflammatory skin conditions [16]. Another health and antiaging benefit of oat Avns is their antigenotoxic activity, which can protect the DNA of epidermal cells against environmental insults, including UV irradiation [21]. In hair care, oat Avns have been shown to prevent lipid peroxidation in human hair follicles and alleviate scalp itchiness and tenderness, indicating Avns as an ideal active ingredient for scalp care formulations [25]. Furthermore, Avns have been shown to prevent oxidation of LDL cholesterol and inhibit the first stages of atherosclerosis, gaining the reputation of being able to protect the aging cardiovascular system. In addition, as also described in this review, several studies in the past few years have suggested that oat Avns may be beneficial in the treatment of various aging-related human diseases associated with chronic oxidative stress and inflammation [96]. Notably, Avns exert their strong antioxidant and anti-inflammatory properties even at very low doses.

Taken together, the established beneficial effects of Avns in skin protection and treatment of dermatological diseases, and their emerging potentiality to prevent and treat chronic oxidative stress and inflammation associated with onset, progression and severity of aging-related diseases, including metabolic, cardiovascular, cerebrovascular and neurodegenerative diseases, point to these compounds as promising new elixir of youth with both cosmetic and pharmaceutical applications.

## 8. Foodomics for Elucidating Molecular Pathways Underlying Biological Effects of Avenanthramides in Chronic Diseases

Nutrition research has traditionally explored the functional importance of diverse food categories through a careful evaluation of various physiological phenomena and molecular markers characterizing a group of individuals fed with a defined diet. Bioactive food constituents may have significant beneficial effects for health promotion and disease prevention, with various compounds active in reducing the sustained oxidative stress and inflammation accompanying chronic diseases, for example, CVDs and/or metabolic syndromes. Unfortunately, chronic disorders are often complex, multifactorial pathogenetic processes; they are the result of combined genomic variant peculiarities interacting with environmental/behavioral factors. Hence, not only genetic factors but also homeostatic alterations related to the environment may be crucial in disease onset, progression, and severity.

In the last few years, nutrition research has moved from classical physiology and epidemiology to chemical biology, molecular biology, and genetics [97]. It has evolved similarly to pharmacological research, where the topic effect of a specific drug is evaluated on a defined cellular/organism model subjected to controlled perturbative events (such as drug treatment at a specific concentration and for a defined time), which are then assayed according to a holistic perspective through combined molecular approaches [98, 99]. In this context, foodomics has emerged as a novel and multidisciplinary research field in nutrition science, which aims at elucidating how diet can influence organism health [100, 101]. It is well known that bioactive compounds present in foods, when assimilated, can affect gene expression profiles in organism tissues/organs, and corresponding protein levels and metabolite representations, thus contributing to modulating the incidence of several chronic diseases. The study of these complex interactions requires the integration of different analytical approaches generating various dataset, which then are interpreted according to a system biology perspective by dedicated bioinformatic methods [102]. Thus, in a foodomics experiment, (i) genomics takes advantage of DNA microarray technologies to detect mRNA expression changes in response to diet; (ii) proteomics uses quantitative LC and MS methods combined with isotopic labelling procedures (TMT, iTRAQ, or SILAC) to define protein profile variations in dietary interventions; (iii) metabolomics uses the same separation and measure techniques to define the bioavailability of bioactive molecules in food and their molecular changes after ingestion, as well as organism plasma/urine metabolite profiles in response to diet; (iv) genetics defines common genetic variants involved in the individual response to diet through whole genome sequencing techniques. Integration of all information according to a multiomic elaboration allows simultaneously deciphering gene expression pathways, protein levels, and metabolite concentrations that are affected in healthy individuals experiencing a certain diet; the same information can be obtained for subjects suffering a certain pathological condition. Thus, it is possible to formulate dietary recommendations based on a system biology perspective to ensure a healthy condition or to prevent and treat chronic diseases, such as CVDs, obesity, and cancer [103]. In this context, we particularly underline the importance of foodomics studies that over time have been performed on human, animals, and animal models of human diseases administered with (i) rosemary extracts rich in polyphenols [104–106] and corresponding isolated metabolite carnosol and carnosic acid [107, 108]; (ii) red-to-blue fruit extracts rich in anthocyanins [109]; (iii) vegetable extracts rich in flavonoids [110] and isoflavones or isolated genistein, daidzein [111], and flavone [112]; (iv) green tea extracts rich in polyphenols [113]; (v) olive oil extracts rich in polyphenols [114]; (vi) fish oil extracts rich in polyunsaturated fatty acids [115]; (vii) resveratrol-containing foods [116]; (viii) inulin-containing prebiotics and isolated inulin; (ix) increased dietary protein [117, 118]; (x) nutrients lacking normal Zn supplement [119]; (xi) augmented folate [120] and multivitamin/mineral supplement [121]. Due to the complexity of these studies, their results were often published in different articles. In most cases, experiments were performed on individuals fed with a food matrix containing various bioactive compounds; this is similar to other “omic” investigations, where traditional pharmacological remedies were tested through holistic approaches [122–124]. In the next future, it is desirable that advanced foodomics studies, analogous to those reported above for other foods, will also be performed on organisms or animal models of human diseases fed with oat compounds, including isolated Avns and their recombinant derivatives, to unveil the molecular mechanisms underlying the corresponding biological effects and therapeutic benefits reported above. To this regard, particular attention should be paid to the effects of Avns on the intestinal microbiome, as this has been recognized as a fundamental player in human health and disease, affecting a variety of conditions such as host energy balance and immune responses [125], and has been recently implicated also in the pathogenesis of CCM disease, suggesting that manipulation of the bacterial microbiome may indeed be an effective therapeutic approach [126]. It is therefore important that future foodomics investigations will also include information from gut-residing bacteria and consequent modulation of the gut-brain axis.

## 9. Redox Proteomics for Detailing Chemical Modifications Hampered by Avenanthramides in Chronic Diseases

Oxidative and nitrosative stresses, due to an imbalance between the generation of ROS and reactive nitrogen species (RNS), and the antioxidant defense capacity of the organism, are important pathophysiological events contributing to the onset and progression of several human pathologies, including cardiovascular diseases and metabolic syndromes [127, 128]. ROS include superoxide anion (O_2_^•−^), hydroxyl (OH^•^) and peroxyl (RO_2_^•^), and alkoxyl (RO^•^) radicals, as well as nonradical compounds, such as hydrogen peroxide (H_2_O_2_), hypochlorous acid (HOCl) and organic peroxides, which can be produced from either endogenous (e.g., mitochondrial electron transport chain, cytochrome P450 monooxygenases, and NADPH oxidases) or exogenous sources (e.g., pollutants, drugs, xenobiotics, and radiation). On the other hand, RNS are reactive compounds derived from nitric oxide (NO^•^) following the activity of inducible nitric oxide synthases, and include peroxynitrite (ONOO^−^), alkyl peroxynitrite (ROONO), nitrogen dioxide (NO_2_^•^), and other molecules [129].

ROS and RNS affect major cellular components, including lipids, DNA and proteins, modifying their structure. In particular, hundreds of adducts of distinct nature have been identified in proteins as a result of the reaction of ROS and RNS with chemical groups present in amino acid side chain [130]. Through modulation of protein structure/function, ROS and RNS can influence a number of enzymatic activities and protein functions, thus affecting intracellular signal transduction pathways and gene expression profiles. While several enzymatic and nonenzymatic markers of chronic oxidative and nitrosative stresses are well known in different organs and body tissues/fluids, early protein targets of oxidative and nitrosative injuries are now becoming to be defined. The identification of putative oxidative biomarkers takes advantage of redox proteomics [131], which is indeed a branch of proteomics specifically designed to identify oxidized and nitrosized proteins and determine nature, extent, and location of oxidative/nitrosative posttranslational modifications in the proteomes of interest. *Gel-based* and *gel-free* redox proteomics techniques often use liquid chromatography coupled to mass spectrometry as the major platform to achieve the goal of identifying and fully characterizing oxidized and nitrosized target proteomes. In this context, dedicated redox proteomics methods have been developed to quali-quantitatively investigate (i) Cys oxidation to sulfenic, sulfinic, and sulfonic acid; (ii) Cys conversion to intra- and intermolecular cystine derivatives; (iii) Cys conversion into S-nitrosyl-cysteine; (iv) Met sulfoxidation to sulfone/sulfoxide derivatives; (v) Trp oxidation and nitrosation to (di)hydroxytryptophan, N-formylkynurenine, hydroxykynurenine, kynurenine, and nitrotryptophan; (vi) His oxidation to oxindolylalanine, 2-oxo-histidine, and 5-hydroxy-2-oxo-histidine; (vii) Tyr oxidation, nitrosation, and halogenation to di- and tri-hydroxyphenylalanine, 3,3′-dityrosine, 3-nitrotyrosine, and 3,(5)-(di)halotyrosine, respectively; (viii) Pro, Arg, Lys, and Thr direct oxidation to 2-pyrrolidone, glutamic acid semialdehyde, aminoadipic semialdehyde, and 2-amino-3-ketobutyric acid, respectively; (ix) Lys and Arg glyco-oxidation to generate more than fifty distinct derivatives; (x) Cys, His, and Lys reactions with *α*,*β*-unsaturated aldehydes deriving from lipid peroxidation to generate more than thirty distinct derivatives; (xi) Cys modification with electrophic prostaglandins and isoprostanes deriving from arachidonic acid oxidation. Once identified, oxidized and nitrosized proteins can be placed in specific molecular pathways to provide insights into affected molecular and cellular functions associated with human diseases.

Conventional and early detection of above-mentioned oxidized and nitrosized protein markers in various diseases and metabolic syndromes has thus enabled to hypothesize a relationship between pathological hallmarks of such disorders and protein structural/functional modifications. This is the case of distinctive identifications of (i) carbonylation, Tyr chlorination, and Met sulfoxidation of target proteins in plasma and atherosclerotic lesions from subjects affected by coronary artery diseases [132, 133]; (ii) glycated, carbonylated, Met-sulfoxidized, Tyr-nitrated, and *S-*nitrosylated proteins in biological fluids and tissues of diabetic patients, or tissues of related animal models [134–144]; (iii) carbonylation, Tyr nitration, and *S-*glutathionylation of target proteins in brain tissues of AD and mild cognitive impairment patients, or animal models of AD, PD, HD, and ALS [145–150]; (iv) oxidized, carbonylated, and Tyr-nitrated and chlorinated proteins in body tissues of patients and animal models experiencing various acute inflammatory syndromes [151, 152]; (v) carbonylated proteins in bronchoalveolar lavage of patients with sarcoidosis and pulmonary fibrosis [153]; (vi) carbonylated proteins in the diaphragm and muscle tissues of severe chronic obstructive pulmonary disease patients and related animal models [154, 155]; (vii) glycoxidized and carbonylated proteins in urine and the kidney of patients with dialysis-related amyloidosis [156–158]; **(**viii) oxidized proteins in human liver tissues after ischemia/reperfusion [159]; (ix) redox modified proteins in various tissues as a result of aging [160–168]; (x) carbonylated, Tyr-nitrated, and S-sulphenylated proteins in hypertensive kidney disease [169–171]; (xi) oxidized proteins in the amniotic fluid of preeclamptic women [172]. Whenever inserted in perturbative experiment pipelines, redox proteomics approaches are now allowing a monitoring of the degree of corresponding body tissue damage and the response to pharmacological therapies.

At the same time, they will provide a rationale to the positive/negative effects of a diet on healthy individuals and/or on patients suffering pathological conditions. In this context, pioneering experiments have been performed to evaluate the impact of (i) excessive caloric intake on oxidized and carbonylated proteins from adipose tissues of healthy men [173]; (ii) fasting on Cys-oxidized proteins from healthy animals [174]; (iii) high-fat and high-sucrose diet on carbonylated proteins from tissues and body fluids of healthy animals [175]; (iv) the assumption of glutathione derivatives on Tyr-nitrated proteins from brain-injured animal models [176]; (v) antioxidant-fortified diet on carbonylated and Tyr-nitrated proteins from brain tissues of animal models of AD [177]; (vi) high-fat and alcohol diet on carbonylated and Cys-oxidized proteins from tissues and body fluids of fatty liver disease patients and related animal models [178–184]. In the close future, it is hypothesized that redox proteomics studies will also be performed on organisms or animal models of human diseases fed with oat compounds and derivatives, including isolated natural and recombinant Avns, in order to evaluate the capacity of such nutraceuticals to modulate oxidized and nitrosized proteomes in target tissues and body fluids.

## 10. Concluding Remarks

It is generally accepted that antioxidants exert health-promoting effects by scavenging intracellular ROS; thus, their consumption as food additives and nutraceuticals has been greatly encouraged. Nonetheless, to date, there is little clinical evidence for the long-term benefits of most antioxidants, while there are even alarms of health risks consequent to supplementation of lipophilic antioxidants [68]. Accordingly, the existence of a physiological role of specific ROS concentrations can explain the negative results from clinical trials, where large doses of exogenously-administered antioxidants or hyperactivation of antioxidant pathways with electrophilic therapeutics failed to improve outcomes of oxidative stress-related diseases or resulted even deleterious [63, 67, 185, 186]. Indeed, it is now well established that redox reactions bear the Janus faceted feature of promoting both physiological signaling responses and pathological cues in all biological systems, as well as that endogenous antioxidant molecules and mechanisms participate in both scenarios [63, 67]. Consistently, emerging evidence demonstrates that only intermediate levels of major regulators of antioxidant responses are beneficial, although both the low and high concentration thresholds for physiological versus pathological effects may vary largely depending on genetic and environmental factors and the cellular context [185]. Thus, given that most of the emerging therapeutic compounds with antioxidant properties influence redox-sensitive mechanisms, both their low and high concentration thresholds for physiological versus pathological effects have to be carefully considered.

**Figure 2:**
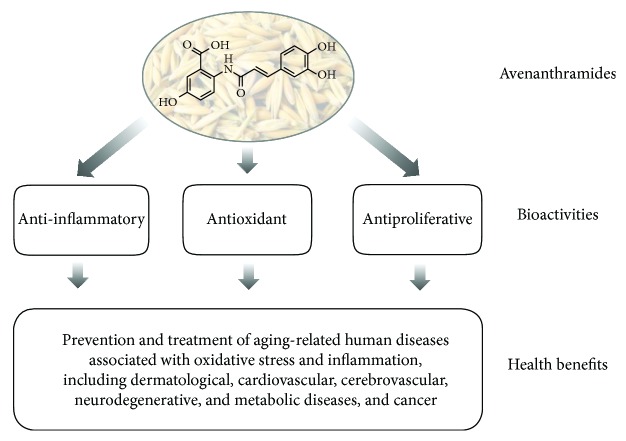
Bioactivities and potential health benefits of avenanthramides. Both natural, synthetic, and recombinant avenanthramides have been shown to exhibit strong antioxidant, anti-inflammatory, and antiproliferative activities, which may provide protection against various cellular dysfunctions and human pathologies, including aging-related diseases.

In this light, further studies are necessary to fully address the beneficial effects of Avns in human health, including antioxidant, antiproliferative, anti-inflammatory, antiaging, and anticancer activities (Figure 2). In particular, useful insights could be derived from foodomics and redox proteomics studies aimed at a comprehensive characterization of molecules and mechanisms that mediate the pleiotropic effects of Avns in cellular and animal models of human diseases, including oxidative posttranslational modifications of structural and regulatory proteins. Moreover, novel therapeutic approaches, including combinatorial therapy and nanotechnology-based targeted drug delivery, are encouraged in order to allow site-directed application, appropriate dosing regimens, pharmacological repair of oxidized biomolecules, and triggering of endogenous antioxidant response systems, which could also be guided by the identification of predictive biomarkers.

## Figures and Tables

**Table 1:** Antioxidant activity of natural, synthetic, and recombinant avenanthramides.

Year	Compound	Effects	Ref.
1937	Oat flour	Food preservation from oxidative deterioration	[42, 43]
1987	Tranilast	Reduction of intracellular levels of ROS, including hydrogen peroxide (H_2_O_2_) and hydroxyl radical (OH^•^)	[51]
2003	Avns	Antioxidant activity demonstrated by using DPPH (2,2-diphenyl-1-picrylhydrazyl), FRAP (ferric reducing antioxidant potential), and linoleic acid assays	[20, 24, 28]
2003	Avn-C	Upregulation of superoxide dismutase and glutathione peroxidase activities and attenuation of exercise-induced ROS production and lipid peroxidation in the heart and skeletal muscles of rats	[19]
2004	Supplementation of Avn-enriched extract of oats	Interaction with vitamin C to enhance hamster and human LDL resistance to oxidation	[22]
2007	Consumption of Avn-enriched extract of oats	Antioxidant activity in humans: increase of the plasma-reduced glutathione level after consumption	[21]
2010	Avn-rich extract from oat	Effective against D-galactose-induced oxidative stress	[48]
2010	YAvns	Reduction of intracellular ROS levels in a cellular model of CCM disease	[39]
2015	YAvns	Upregulation of FOXO1 and SOD2 expressions in a cellular model of CCM disease	[40]
2015	Avns	Upregulation of heme oxygenase-1 (HO-1) expression in both a dose- and time-dependent manner mediated by Nrf2 translocation	[50]
2017	YAvns	Antioxidant effects in a mouse model of CCM disease	[41]
2018	Natural and synthetic Avns	Antioxidant effects on CaCo-2 and Hep3B cancer cells	[83]

Avns: avenanthramides; FOXO1: forkhead box protein O1; ROS: reactive oxygen species; SOD2: superoxide dismutase 2; YAvns: yeast avenanthramides.

**Table 2:** Anti-inflammatory activity of natural, synthetic, and recombinant avenanthramides.

Year	Compound	Effects	Ref.
1997	Tranilast	Inhibition of COX-2 and iNOS expression	[56]
2002	Tranilast	Inhibition of cytokine-induced NF-*κ*B activation	[16]
2004	Avn-enriched extract of oats	Inhibition of IL-6, IL-8, and MCP-1 secretion and ICAM-1, VCAM-1, and E-selectin expression	[29]
2008	CH_3_-Avn-C	Reduction of mRNA expression and secretion of IL-6, IL-8, and MCP-1 and inhibition of IL-1*β*- and TNF*α*-stimulated NF-*κ*B activation in endothelial cells	[26]
2008	Avns	Inhibition of TNF-induced NF-*κ*B activity and reduction of IL-8 release keratinocites. Putative anti-itching activity	[29]
2008	Avns	Inhibition of tumor necrosis factor alpha (TNF-alpha) induced NF-*κ*B luciferase activity and subsequent reduction of interleukin-8 (IL-8)	[16]
2014	Avn-based diet supplementation	Attenuation of exercise-induced inflammation	[53]
2015	Avn-enriched oat bran	Modulation of specific biomarkers of inflammation in older, overweight, or obese adults	[82]
2017	YAvns	Inhibition of NF-*κ*B and rescue of inflammatory phenotypes in cellular and mouse models of CCM disease	[41]
2017	DH Avn-D	Interaction with the neurokinin-1 receptor (NK1R), inhibition of mast cell degranulation, and reduction of the secretion of the cytokine interleukin-6 (IL-6)	[52]
2018	Natural and synthetic Avns	Anti-inflammatory effects on CaCo-2 and Hep3B cancer cells	[83]

Avns: avenanthramides; CCM: cerebral cavernous malformation; CH_3_-Avn-C: methyl ester of Avn-C; COX-2: cyclooxygenase-2; DH Avn-D: dihydro-avenanthramide D; ICAM-1: intercellular adhesion molecule 1; iNOS: inducible nitric oxide synthase; MCP-1: monocytic chemotactic protein-1; NF-*κ*B: nuclear factor kappa-light-chain-enhancer of activated B cells; TNF*α*: tumor necrosis factor alpha; VCAM-1: vascular cell adhesion molecule 1; YAvns: yeast avenanthramides.

**Table 3:** Antiproliferative activity of natural, synthetic, and recombinant avenanthramides.

Year	Compound	Effects	Ref.
1994–1996	Tranilast	Blockage of PDGF-induced cell-cycle progression at the G1/S checkpoint, inhibition of VSMC proliferation, and suppression of intimal hyperplasia after photochemically induced endothelial injury in the rat	[31]
1994–1997	Tranilast	Proposed as a putative therapeutic agent for prevention and treatment of diseases associated with neovascularization, such as diabetic retinopathy, senile discoid macular degeneration, neovascular glaucoma, and rheumatoid arthritis	[59–62]
2001	Tranilast	Inhibition of migration and invasiveness of human malignant glioma cells	[37]
2002	Tranilast	Inhibition of pancreatic cancer cell proliferation and tumor angiogenesis	[58]
2003	Tranilast	Inhibition of oral squamous cell carcinoma growth and invasion	[76]
2006	Avn-C and CH_3_-Avn-C	Inhibition of VSMC proliferation	[31]
2006	Avn-C	Inhibition of SMC proliferation by upregulating the p53-p21cip1 pathway and inhibiting pRB phosphorylation	[30, 31]
2009	Tranilast	Inhibition of human prostate adenocarcinoma cell proliferation	[74]
2009	Tranilast	Inhibition of neurofibroma cell growth	[81]
2010	Tranilast	Effectiveness in the treatment of desmoid tumor of the chest wall and inhibition of breast cancer stem cells	[73]
2010	Tranilast	Inhibition of murine and human breast cancer cell proliferation and migration	[79, 80]
2010	Avn-enriched extracts of oats, Avn-C, and CH_3_-Avn-C	Antiproliferative effects on distinct colon cancer cell lines	[25]
2011	DH Avn-D	Inhibition of human breast cancer cell invasion through downregulation of MAPK/NF-*κ*B and MAPK/AP-1 pathways and suppression of MMP-9 expression	[32]
2015	YAvns	Stronger antiproliferative properties than natural Avns, including Avn-B, due to enhanced capacity of reducing intracellular ROS levels and cyclin D1 expression	[40]
2017	Avns	Antiproliferative effect on breast cancer cells through an antiapoptotic mechanism as revealed by annexin V and caspase activities	[57]
2018	Natural and synthetic Avns	Cytotoxic and proapoptotic effects on CaCo-2 and Hep3B cancer cells	[83]

Avns: avenanthramides; CH_3_-Avn-C: methyl ester of Avn-C; DH Avn-D: dihydro-avenanthramide D; PDGF: platelet-derived growth factor; ROS: reactive oxygen species; VSMC: vascular smooth muscle cells; YAvns: yeast avenanthramides.

## Abbreviations

Avn: Avenanthramide
CCM: Cerebral cavernous malformations
CHD: Coronary heart disease
COX-2: Cyclooxygenase-2
FOXO1: Forkhead box protein O1
HO-1: Heme oxygenase-1
ICAM-1: Intercellular adhesion molecule 1
I*κ*B: Nuclear factor of kappa light polypeptide gene enhancer in B cells inhibitor
LDL: Low-density lipoprotein
MMP: Matrix metalloproteinase
NF-*κ*B: Nuclear factor kappa-light-chain-enhancer of activated B cells
VCAM-1: Vascular cell adhesion molecule 1
ROS: Reactive oxygen species
SOD2: Superoxide dismutase 2
TGF-*β*: Transforming growth factor-*β*
TNF*α*: Tumor necrosis factor *alpha*
VSMC: Vascular smooth muscle cells
YAvn: Yeast avenanthramide.
